# Supplementary material for: Pharmacological dimerization and activation of the exchange factor eIF2B antagonizes the integrated stress response
Source: eLife. 2015 Apr 15;4:e07314. doi: 10.7554/eLife.07314 (PMC4426669; doi:10.7554/eLife.07314)
Supplement: Figure 1—source data 1. — DOI: http://dx.doi.org/10.7554/eLife.07314.004 [file elife07314s001.pdf]

<Serial Cloner V2.5>
Map of pMK1163

tgagcaaaaacaggaaggcaaaatgccgcaaaaaagggaataagggcgacacggaaatgttgaatactcatactcttcctttttcaatattattgaagca < 100

<Amp prom

tttatcagggttattgtctcatgagcggatacatatttgaatgtatttagaaaaataaacaaataggggttccgcgcacatttccccgaaaagtgccacc < 200

tgacgcttaagcggtcgacggatcgggagatctcccgatcccctatggtgcactctcagtacaatctgctctgatgccgcatagttaagccagtatctgc < 300

tccctgcttgtgtgttggaggtcgctgagtagtgcgcgagcaaaatttaagctacaacaaggcaaggcttgaccgacaattgcatgaagaatctgcttag < 400

ggttaggcgttttgcgctgcttcgcgatgtacgggccagatatacgcgttgacattgattattgactagttattaatagtaatcaattacggggtcatta < 500

gttcatagcccatatatggagttccgcgttacataacttacggtaaatggcccgcctggctgaccgcccaacgacccccgcccattgacgtcaataatga < 600

cgtatgttcccatagtaacgccaatagggactttccattgacgtcaatgggtggagtatttacggtaaactgcccacttggcagtacatcaagtgtatca < 700

tatgccaagtacgccccctattgacgtcaatgacggtaaatggcccgcctggcattatgcccagtacatgaccttatgggactttcctacttggcagtac < 800

atctacgtattagtcatcgctattaccatggtgatgcggttttggcagtacatcaatgggcgtggatagcggtttgactcacggggatttccaagtctcc < 900

accccattgacgtcaatgggagtttgttttggcaccaaaatcaacgggactttccaaaatgtcgtaacaactccgccccattgacgcaaatgggcggtag < 1000

gcgtgtacggtgggaggtctatataagcagcgcgttttgcctgtactgggtctctctggttagaccagatctgagcctgggagctctctggctaactagg < 1100

>HIV-1 5 LTR

gaacccactgcttaagcctcaataaagcttgccttgagtgcttcaagtagtgtgtgcccgtctgttgtgtgactctggtaactagagatccctcagaccc < 1200

ttttagtcagtgtggaaaatctctagcagtggcgcccgaacagggacttgaaagcgaaagggaaaccagaggagctctctcgacgcaggactcggcttgc < 1300

>HIV-1 psi pack

tgaagcgcgcacggcaagaggcgaggggcggcgactggtgagtacgccaaaaattttgactagcggaggctagaaggagagagatgggtgcgagagcgtc < 1400

agtattaagcgggggagaattagatcgcgatgggaaaaaattcggttaaggccagggggaaagaaaaaatataaattaaaacatatagtatgggcaagca < 1500

gggagctagaacgattcgcagttaatcctggcctgttagaaacatcagaaggctgtagacaaatactgggacagctacaaccatcccttcagacaggatc < 1600

agaagaacttagatcattatataatacagtagcaaccctctattgtgtgcatcaaaggatagagataaaagacaccaaggaagctttagacaagatagag < 1700

gaagagcaaaacaaaagtaagaccaccgcacagcaagcggccggccgcgctgatcttcagacctggaggaggagatatgagggacaattggagaagtgaa < 1800

ttatataaatataaagtagtaaaaattgaaccattaggagtagcacccaccaaggcaaagagaagagtggtgcagagagaaaaaagagcagtgggaatag < 1900

gagctttgttccttgggttcttgggagcagcaggaagcactatgggcgcagcgtcaatgacgctgacggtacaggccagacaattattgtctggtatagt < 2000

>RRE

gcagcagcagaacaatttgctgagggctattgaggcgcaacagcatctgttgcaactcacagtctggggcatcaagcagctccaggcaagaatcctggct < 2100

gtggaaagatacctaaaggatcaacagctcctggggatttggggttgctctggaaaactcatttgcaccactgctgtgccttggaatgctagttggagta < 2200

ataaatctctggaacagatttggaatcacacgacctggatggagtgggacagagaaattaacaattacacaagcttaatacactccttaattgaagaatc < 2300

gcaaaaccagcaagaaaagaatgaacaagaattattggaattagataaatgggcaagtttgtggaattggtttaacataacaaattggctgtggtatata < 2400

aaattattcataatgatagtaggaggcttggtaggtttaagaatagtttttgctgtactttctatagtgaatagagttaggcagggatattcaccattat < 2500

cgtttcagacccacctcccaaccccgaggggacccgacaggcccgaaggaatagaagaagaaggtggagagagagacagagacagatccattcgattagt < 2600

gaacggatcggcactgcgtgcgccaattctgcagacaaatggcagtattcatccacaattttaaaagaaaaggggggattggggggtacagtgcagggga < 2700

aagaatagtagacataatagcaacagacatacaaactaaagaattacaaaaacaaattacaaaaattcaaaattttcgggtttattacagggacagcaga < 2800

gatccagtttggttagtaccgggcccgctctagagatccgacgcgccatctctaggcccgcgccggcccccctcgcacagacttgtgggagaagctcggct < 2900

actcccctgccccggttaatttgcatataatatttcctagtaactatagaggcttaatgtgcgataaaagacagataatctgttctttttaatactagct < 3000

>LoxP

acattttacatgataggcttggatttctataacttcgtatagcatacattatacgaagttataaacagcacaaaaggaaactcaccctaactgtaaagta < 3100

attgtgtgttttgagactataaatatcccttggagaaaagccttgttaacgcgcggtgaccctcgagtactaggatccattaggcggccgcgtggataac < 3200

cgtattaccgccatgcattagttattaATAGTAATCAATTACGGGGTCATTAGTTCATAGCCCATATATGGAGTTCCGCGTTACATAACTTACGGTAAAT < 3300

GGCCCGCCTGGCTGACCGCCCAACGACCCCCGCCCATTGACGTCAATAATGACGTATGTTCCCATAGTAACGCCAATAGGGACTTTCCATTGACGTCAAT < 3400

GGGTGGAGTATTTACGGTAAACTGCCCACTTGGCAGTACATCAAGTGTATCATATGCCAAGTACGCCCCCTATTGACGTCAATGACGGTAAATGGCCCGC < 3500

>P-CMV

CTGGCATTATGCCCAGTACATGACCTTATGGGACTTTCCTACTTGGCAGTACATCTACGTATTAGTCATCGCTATTACCATGGTGATGCGGTTTTGGCAG < 3600

TACATCAATGGGCGTGGATAGCGGTTTGACTCACGGGGATTTCCAAGTCTCCACCCCATTGACGTCAATGGGAGTTTGTTTTGGCACCAAAATCAACGGG < 3700

ACTTTCCAAAATGTCGTAACAACTCCGCCCCATTGACGCAAATGGGCGGTAGGCGTGTACGGTGGGAGGTCTATATAAGCAGAGCTGGTTTAGTGAACCG < 3800

TCAGATCCGCTAGCGCTACCGGACTCAGATCTCGAGtttctactttgcccgcccacagatgtagttttctctgcgcgtgtgcgttttccctcctccccgc < 3900

>ATF4-UTRa

cctcagggtccacggccaccatggcgtattaggggcagcagtgcctgcggcagcattggcctttgcagcggcggcagcagcaccaggctctgcagcggca < 4000

acccccagcggcttaagccatggcgcttctcacggcattcagcagcagcgttgctgtaaccgacaaagacaccttcgaattaagcacattcctcgattcc < 4100

agcaaagcaccgcaacATGGTGAGCAAGGGCGAGGAGCTGTTCACCGGGGTGGTGCCCATCCTGGTCGAGCTGGACGGCGACGTAAACGGCCACAAGTTC < 4200

AGCGTGTCCGGCGAGGGCGAGGGCGATGCCACCTACGGCAAGCTGACCCTGAAGCTGATCTGCACCACCGGCAAGCTGCCCGTGCCCTGGCCCACCCTCG < 4300

TGACCACCCTGGGCTACGGCCTGCAGTGCTTCGCCCGCTACCCCGACCACATGAAGCAGCACGACTTCTTCAAGTCCGCCATGCCCGAAGGCTACGTCCA < 4400

>Venus

GGAGCGCACCATCTTCTTCAAGGACGACGGCAACTACAAGACCCGCGCCGAGGTGAAGTTCGAGGGCGACACCCTGGTGAACCGCATCGAGCTGAAGGGC < 4500

ATCGACTTCAAGGAGGACGGCAACATCCTGGGGCACAAGCTGGAGTACAACTACAACAGCCACAACGTCTATATCACCGCCGACAAGCAGAAGAACGGCA < 4600

TCAAGGCCAACTTCAAGATCCGCCACAACATCGAGGACGGCGGCGTGCAGCTCGCCGACCACTACCAGCAGAACACCCCCATCGGCGACGGCCCCGTGCT < 4700

GCTGCCCGACAACCACTACCTGAGCTACCAGTCCGCCCTGAGCAAAGACCCCAACGAGAAGCGCGATCACATGGTCCTGCTGGAGTTCGTGACCGCCGCC < 4800

GGGATCACTCTCGGCATGGACGAGCTGTACAAGTAAGAATTCCGCCCCTCTCCCTCCCCCCCCCCTAACGTTACTGGCCGAAGCCGCTTGGAATAAGGCC < 4900

GGTGTGCGTTTGTCTATATGTTATTTTCCACCATATTGCCGTCTTTTGGCAATGTGAGGGCCCGGAAACCTGGCCCTGTCTTCTTGACGAGCATTCCTAG < 5000

GGGTCTTTCCCCTCTCGCCAAAGGAATGCAAGGTCTGTTGAATGTCGTGAAGGAAGCAGTTCCTCTGGAAGCTTCTTGAAGACAAACAACGTCTGTAGCG < 5100

>IRES

ACCCTTTGCAGGCAGCGGAACCCCCCACCTGGCGACAGGTGCCTCTGCGGCCAAAAGCCACGTGTATAAGATACACCTGCAAAGGCGGCACAACCCCAGT < 5200

GCCACGTTGTGAGTTGGATAGTTGTGGAAAGAGTCAAATGGCTCTCCTCAAGCGTATTCAACAAGGGGCTGAAGGATGCCCAGAAGGTACCCCATTGTAT < 5300

GGGATCTGATCTGGGGCCTCGGTGCACATGCTTTACATGTGTTTAGTCGAGGTTAAAAAACGTCTAGGCCCCCCGAACCACGGGGACGTGGTTTTCCTT < 5400

TGAAAAACACGATGATAATATGGCCACAACCATGAGCGAGCTGATTAAGGAGAACATGCACATGAAGCTGTACATGGAGGGCACCGTGGACAACCATCAC < 5500

TTCAAGTGCACATCCGAGGGCGAAGGCAAGCCCTACGAGGGCACCCAGACCATGAGAATCAAGGTGGTCGAGGGCGGCCCTCTCCCCTTCGCCTTCGACA < 5600

TCCTGGCTACTAGCTTCCTCTACGGCAGCAAGACCTTCATCAACCACACCCAGGGCATCCCCGACTTCTTCAAGCAGTCCTTCCCTGAGGGCTTCACATG < 5700

>tagBFP

GGAGAGAGTCACCACATACGAAGACGGGGGCGTGCTGACCGCTACCCAGGACACCAGCCTCCAGGACGGCTGCCTCATCTACAACGTCAAGATCAGAGGG < 5800

GTGAACTTCACATCCAACGGCCCTGTGATGCAGAAGAAAACACTCGGCTGGGAGGCCTTCACCGAGACGCTGTACCCCGCTGACGGCGGCCTGGAAGGCA < 5900

GAAACGACATGGCCCTGAAGCTCGTGGGCGGGAGCCATCTGATCGCAAACATCAAGACCACATATAGATCCAAGAAACCCGCTAAGAACCTCAAGATGCC < 6000

TGGCGTCTACTATGTGGACTACAGACTGGAAAGAATCAAGGAGGCCAACAACGAGACCTACGTCGAGCAGCACGAGGTGGCAGTGGCCAGATACTGCGAC < 6100

CTCCCTAGCAAACTGGGGCACAAGCTTAATTAAgcctaggcGAATTCgtcgagggacctaataacttcgtatagcatacattatacgaagttatacatgt < 6200

ttaagggttccggttccactaggtacaattcgatatcaagcttatcgataatcaacctctggattacaaaatttgtgaaagattgactggtattcttaac < 6300

tatgttgctccttttacgctatgtggatacgctgctttaatgcctttgtatcatgctattgcttcccgtatggctttcattttctcctccttgtataaat < 6400

cctggttgctgtctctttatgaggagttgtggcccgttgtcaggcaacgtggcgtggtgtgcactgtgtttgctgacgcaacccccactggttggggcat < 6500

tgccaccacctgtcagctcctttccgggactttcgctttccccctccctattgccacggcggaactcatcgccgcctgccttgcccgctgctggacaggg < 6600

gctcggctgttgggcactgacaattccgtggtgttgtcggggaaatcatcgtcctttccttggctgctcgcctgtgttgccacctggattctgcgcggga < 6700

cgtccttctgctacgtcccttcggccctcaatccagcggaccttccttcccgcggcctgctgccggctctgcggcctcttccgcgtcttcgccttcgccc < 6800

tcagacgagtcggatctccctttgggccgcctccccgcatcgataccgtcgacctcgatcgagacctagaaaaacatggagcaatcacaagtagcaatac < 6900

agcagctaccaatgctgattgtgcctggctagaagcacaagaggaggaggaggtgggttttccagtcacacctcaggtacctttaagaccaatgacttac < 7000

aaggcagctgtagatcttagccactttttaaaagaaaaggggggactggaagggctaattcactcccaacgaagacaagatatccttgatctgtggatct < 7100

accacacacaaggctacttccctgattggcagaactacacaccagggccagggatcagatatccactgacctttggatggtgctacaagctagtaccagt < 7200

tgagcaagagaaggtagaagaagccaatgaaggagagaacacccgcttgttacaccctgtgagcctgcatgggatggatgacccggagagagaagtatta < 7300

gagtggaggtttgacagccgcctagcatttcatcacatggcccgagagctgcatccggactgtactgggtctctctggttagaccagatctgagcctggg < 7400

>HIV-1 5 LTR

agctctctggctaactagggaacccactgcttaagcctcaataaagcttgccttgagtgcttcaagtagtgtgtgcccgtctgttgtgtgactctggtaa < 7500

ctagagatccctcagacccttttagtcagtgtggaaaatctctagcagcatgtgagcaaaaggccagcaaaaggccaggaaccgtaaaaaggccgcgttg < 7600

ctggcgtttttccataggctccgcccccctgacgagcatcacaaaaatcgacgctcaagtcagaggtggcgaaacccgacaggactataaagataccagg < 7700

cgtttccccctggaagctccctcgtgcgctctcctgttccgaccctgccgcttaccggatacctgtccgcctttctcccttcgggaagcgtggcgctttc < 7800

tcatagctcacgctgtaggtatctcagttcggtgtaggtcgttcgctccaagctgggctgtgtgcacgaaccccccgttcagcccgaccgctgcgcctta < 7900

<ColE1 origin

tccggtaactatcgtcttgagtccaacccggtaagacacgacttatcgccactggcagcagccactggtaacaggattagcagagcgaggtatgtaggcg < 8000

gtgctacagagttcttgaagtggtggcctaactacggctacactagaagaacagtatttggtatctgcgctctgctgaagccagttaccttcggaaaaag < 8100

agttggtagctcttgatccggcaaacaaaccaccgctggtagcggtggtttttttgtttgcaagcagcagattacgcgcagaaaaaaaggatctcaagaa < 8200

gatcctttgatcttttctacggggtctgacgctcagtggaacgaaaactcacgttaagggattttggtcatgagattatcaaaaaggatcttcacctaga < 8300

tccttttaaattaaaaatgaagttttaaatcaatctaaagtatatatgagtaaacttggtctgacagttaccaatgcttaatcagtgaggcacctatctc < 8400

agcgatctgtctatttcgttcatccatagttgcctgactccccgtcgtgtagataactacgatacgggagggcttaccatctggccccagtgctgcaatg < 8500

ataccgcgagacccacgctcaccggctccagatttatcagcaataaaccagccagccggaagggccgagcgcagaagtggtcctgcaactttatccgcct < 8600

ccatccagtctattaattgttgccgggaagctagagtaagtagttcgccagttaatagtttgcgcaacgttgttgccattgctacaggcatcgtggtgtc < 8700

acgctcgtcgtttggtatggcttcattcagctccggttcccaacgatcaaggcgagttacatgatcccccatgttgtgcaaaaaagcggttagctccttc < 8800

ggtcctccgatcgttgtcagaagtaagttggccgcagtgttatcactcatggttatggcagcactgcataattctcttactgtcatgccatccgtaagat < 8900

gcttttctgtgactggtgagtactcaaccaagtcattctgagaatagtgtatgcggcgaccgagttgctcttgcccggcgtcaatacgggataataccgc < 9000

```
gccacatagcagaactttaaaagtgctcatcattggaaaacgttcttcggggcgaaaactctcaaggatcttaccgctgttgagatccagttcgatgtaa  < 9100

cccactcgtgcacccaactgatcttcagcatcttttactttcaccagcgtttctggg  < 9157
```

```
Features :
P-CMV                     : [3228 : 3780]
IRES                      : [4844 : 5431]
tagBFP                    : [5432 : 6133]
Venus                     : [4117 : 4836]
ATF4-UTRa                 : [3837 : 4116]
ColE1 origin              : [8219 : 7591]
LoxP                      : [3029 : 3062]
Amp prom                  : [141 : 113]
HIV-1 5 LTR               : [1048 : 1228]
HIV-1 5 LTR               : [7367 : 7547]
HIV-1 psi pack            : [1339 : 1383]
RRE                       : [1899 : 2132]
```
